# Rescue of Mutant CFTR Trafficking Defect by the Investigational Compound MCG1516A

**DOI:** 10.3390/cells11010136

**Published:** 2022-01-01

**Authors:** Miquéias Lopes-Pacheco, Mafalda Bacalhau, Sofia S. Ramalho, Iris A. L. Silva, Filipa C. Ferreira, Graeme W. Carlile, David Y. Thomas, Carlos M. Farinha, John W. Hanrahan, Margarida D. Amaral

**Affiliations:** 1 Faculty of Sciences, Biosystems & Integrative Sciences Institute (BioISI), University of Lisbon, 1749-016 Lisbon, Portugal; mrbacalhau@fc.ul.pt (M.B.); ssramalho@fc.ul.pt (S.S.R.); iasilva@fc.ul.pt (I.A.L.S.); fc47772@alunos.fc.ul.pt (F.C.F.); cmfarinha@fc.ul.pt (C.M.F.); msamaral@fc.ul.pt (M.D.A.); 2 Department of Biochemistry, McGill University, Montreal, QC H3G 1Y6, Canada; graeme.carlile@mcgill.ca (G.W.C.); david.thomas@mcgill.ca (D.Y.T.); 3 Department of Physiology, McGill University, Montreal, QC H3G 1Y6, Canada; john.hanrahan@mcgill.ca

**Keywords:** cystic fibrosis, drug discovery, F508del-CFTR, genetic revertants, intestinal organoids, low temperature, protein trafficking, rare mutations

## Abstract

Although some therapeutic progress has been achieved in developing small molecules that correct F508del-CFTR defects, the mechanism of action (MoA) of these compounds remain poorly elucidated. Here, we investigated the effects and MoA of MCG1516A, a newly developed F508del-CFTR corrector. MCG1516A effects on wild-type (WT) and F508del-CFTR were assessed by immunofluorescence microscopy, and biochemical and functional assays both in cell lines and in intestinal organoids. To shed light on the MoA of MCG1516A, we evaluated its additivity to the FDA-approved corrector VX-661, low temperature, genetic revertants of F508del-CFTR (G550E, R1070W, and 4RK), and the traffic-null variant DD/AA. Finally, we explored the ability of MCG1516A to rescue trafficking and function of other CF-causing mutations. We found that MCG1516A rescues F508del-CFTR with additive effects to VX-661. A similar behavior was observed for WT-CFTR. Under low temperature incubation, F508del-CFTR demonstrated an additivity in processing and function with VX-661, but not with MCG1516A. In contrast, both compounds promoted additional effects to low temperature to WT-CFTR. MCG1516A demonstrated additivity to genetic revertant R1070W, while VX-661 was additive to G550E and 4RK. Nevertheless, none of these compounds rescued DD/AA trafficking. Both MCG1516A and VX-661 rescued CFTR processing of L206W- and R334W-CFTR with greater effects when these compounds were combined. In summary, the absence of additivity of MCG1516A to genetic revertant G550E suggests a putative binding site for this compound on NBD1:NBD2 interface. Therefore, a combination of MCG1516A with compounds able to rescue DD/AA traffic, or mimicking the actions of revertant R1070W (e.g., VX-661), could enhance correction of F508del-CFTR defects.

## 1. Introduction

Cystic Fibrosis (CF; OMIM: 219700) is a life-shortening inherited disease caused by mutations in the gene encoding the CF transmembrane conductance regulator (CFTR) protein [1], a cAMP-regulated chloride (Cl^−^) and bicarbonate (HCO_3_^−^) channel expressed at the apical plasma membrane (PM) of secretory epithelia. CF-causing mutations impair CFTR expression, function, and/or stability, leading to abnormal ion transport with alterations in volume and composition of the epithelial surface liquid in several tissues [2,3]. Clinically, the disease is characterized by high sweat Cl^−^ concentration, severe sinopulmonary and gastrointestinal symptoms, pancreatic insufficiency, and male infertility. However, despite its multi-organ involvement, the progressive decline in lung function represents the major cause of morbidity and, ultimately, death in CF [2,3].

CFTR, a member of the ATP-binding cassette (ABC) transporter family, is structurally composed of two homologous halves, each containing a transmembrane domain (TMD1 and TMD2) and a nucleotide-binding domain (NBD1 and NBD2) [4]. The TMDs form the channel pore through which anions may flow along their electrochemical gradient, while NBDs control channel gating by binding and hydrolyzing ATP. An intrinsically disordered and highly charged regulatory domain (RD), which is unique to CFTR, links the two halves of the protein and promotes conformational changes necessary for channel functioning through protein kinase A (PKA) or protein kinase C (PKC) phosphorylation [4].

Among the ~2100 CFTR genetic variants so far reported (http://www.genet.sickkids.on.ca/, accessed on 19 November 2021), only ~400 have confirmed disease liability (http://cftr2.org, accessed on 19 November 2021). However, a single mutation—the deletion of a phenylalanine at position 508 (F508del in NBD1)—is found in at least one allele of 80–85% of individuals with CF worldwide [2]. This mutation impairs NBD1 thermodynamic stability and its interdomain interactions, causing CFTR protein misfolding that is disqualified from exiting the endoplasmic reticulum (ER) by the quality control (ERQC), being, thus, precluded from PM trafficking and targeted for proteasomal degradation [5,6]. Although F508del-CFTR folding is inefficient, it can be, nevertheless, rescued when cells heterologously expressing this mutant are incubated at a low temperature [7,8,9,10] or by CFTR genetic revertants, i.e., second-site mutations in *cis* with F508del that partially rescue this mutant either by correcting folding by filling in its structural pockets (absent in WT-CFTR), or by removing retention signals, thus allowing the protein to evade ERQC [11,12,13,14,15].

Following this proof-of-concept, implementation of high-throughput (HT) screens led to the identification of small-molecule drugs that rescue F508del-CFTR folding and PM trafficking [16]. After medicinal chemistry and clinical testing, these ultimately resulted in the approval of three corrector drugs currently in the clinic for CF: VX-809 (lumacaftor), VX-661 (tezacaftor), or VX-661 plus VX-445 (elexacaftor), all in combination with the gating potentiator VX-770 (ivacaftor) [17,18,19,20]. In clinical trials, either VX-809 or VX-661 plus VX-770 provided therapeutic benefits for individuals with CF carrying two copies of the F508del mutation, although improvements in lung function were relatively modest (3–5 percent predicted forced expiratory volume in 1 sec [ppFEV_1_]) [17,18]. Some of the adverse effects (chest tightness and dyspnea) reported were in clinical trials of VX-809 with VX-770 in F508del-homozygous individuals [17]. However, such effects were not reported in subsequent studies assessing safety and efficacy of VX-661 (chemically similar to VX-809) with VX-770 [18,21]. VX-445 was recently added to the co-treatment with VX-661/VX-770 and conferred greater clinical benefits, including a gain of approximately 10 and 14 ppFEV_1_, respectively, in individuals with CF who were homozygous and heterozygous for the F508del mutation [19,20]. Nevertheless, this triple combination only partially corrects F508del-CFTR folding and trafficking defects [22] and, therefore, individuals taking it may still face multiple complications associated with disease progression, in particular those in the lower end of the benefit curve, with 5% or less in ppFEV_1_ improvement. Long-term studies are in progress to continue monitoring safety and efficacy of these drugs in real-life.

Both experimental and clinical findings have provided strong evidence that corrector combinations are required to achieve F508del-CFTR correction at clinically relevant levels. Nevertheless, other CFTR mutants with folding and trafficking defects, included in class II similarly to F508del [2,23], are not equally responsive to currently available corrector drugs [24,25,26,27,28]. Accordingly, novel correctors from chemically distinct series are under investigation to identify single compounds (or combinations) providing more robust CFTR correction for these rarer variants. In this context, the MCG1516A compound (PubChem ID: 2305758) demonstrated to increase protein stability of purified human F508del-NBD1 in a cellular thermal shift assay and rescued F508del-CFTR processing [29]. However, the mechanism of action (MoA) of MCG1516A and its utility in rescuing other CFTR mutations remains elusive.

Here, we explored the effects of the MCG1516A compound alone or in combination with the FDA-approved corrector drug VX-661 in CF bronchial epithelial (CFBE) cell lines constitutively expressing WT-CFTR or F508del-CFTR. The ability of MCG1516A to rescue F508del-CFTR was further validated in intestinal organoids obtained from individuals with CF (F508del/F508del). The MoA of MCG1516A was investigated by examining its additive effects to low temperature incubation and to previously established CFTR genetic revertants. These included G550E that restores NBD1:NBD2 dimerization [12,14], R1070W that retrieves NBD1:ICL4 interface [13,15], and 4RK (R29K, R516K, R555K, and R766K) that simultaneously withdraws four arginine-framed tripeptides (AFT) retention signals [11,13]. We also assessed the effects of MCG1516A on a CFTR variant lacking the double diacidic code (DD/AA), which is, thus, precluded from exiting the ER via COPII vesicles, although it does not present a major conformational defect [13,30]. Finally, we evaluated whether MCG1516A could rescue other CF-causing mutations, namely G85E, L206W, R334W, T338I, R347P, I507del, V520F, S549R, R560S, M1101K, and N1303K.

## 2. Materials and Methods

### 2.1. Chemicals and Compounds

All reagents were of analytical grade and purchased from commercial sources. Correctors MCG1516A (#STK370345, Vitas-M laboratory, IL, USA) and VX-661 (#S7058, Selleckchem, TX, USA) were diluted in dimethyl sulfoxide (DMSO) and added to cells diluted in 1% fetal bovine serum (FBS, #10270106, Gibco) supplemented antibiotic-free medium. Other reagents (all from Sigma-Aldrich, MO, USA, unless otherwise stated) were also in DMSO solutions: forskolin (Fsk, #66575-29-9), genistein (Gen, #446-72-0), VX-770 (#1144, Selleckchem, Houston, TX, USA). The final concentration of each compound has been indicated in figure legends.

### 2.2. Cell Culture 

CF bronchial epithelial (CFBE) cell lines constitutively expressing CFTR variants (WT, DD/AA, F508del, R560S, or carrying G550E, R1070W, or 4RK in *cis* with F508del) were cultured in Eagle’s minimum essential medium (EMEM, #10-010-CVR, Corning, VA, USA) supplemented with 10% FBS and 2 µg/mL puromycin [14,24]. CFBE cells co-expressing the halide sensitive yellow fluorescence protein (HS-YFP H148Q/I152L) with either WT- or F508del-CFTR were cultured in EMEM supplemented with 10% FBS, and selection antibiotics (0.5 µg/mL puromycin for WT-CFTR-expressing cells and 2 µg/mL puromycin plus 0.6 mg/mL G418 for F508del-expressing ones) [31,32]. CFBE cells stably expressing mCherry-Flag-CFTR variants under the control of a Tet-On promoter were cultured in Dulbecco’s modified Eagle medium (DMEM, #10-013-CV, Corning, VA, USA) supplemented with 10% FBS, 10 µg/mL blasticidin and 2 µg/mL puromycin [33]. Fischer rat thyroid (FRT) cell lines constitutively expressing CFTR mutations were cultured in Coon’s modified F-12-Ham media supplemented with 5% FBS and hygromycin (100 µg/mL). All cells were cultured in a humidified incubator at 5% CO_2_ and 37 °C, except during low temperature experiments, in which cells were maintained at 27 °C for 24 h.

### 2.3. Generation of Novel Cell Lines 

Novel cell lines were generated, as before [24], by lentiviral transduction of parental CFBE cells, which do not express endogenous CFTR. Initially, site-directed mutagenesis was performed to introduce the G85E, L206W, R334W, I507del, or N1303K mutation into CFTR cDNA cloned in the pcDNA5 expression vector. After confirmation by sequencing, the cDNA was re-cloned into the lentiviral expression vector pLVX-Puro and transfected into the HEK-293T cells for production of lentiviral particles. These particles were collected 48 h after transfection and then used to transduce parental CFBE cells. Puromycin (2 µg/mL) was used for selection and efficiency of the transduction was assessed by Western blot (WB) to confirm CFTR expression and processing pattern.

### 2.4. Immunostaining and Fluorescence Microscopy

Twenty-four hours after seeding CFBE cells expressing mCherry-Flag-CFTR variants (WT−, F508del−, DD/AA−, F508del/G550E−, F508del/R1070W−, and F508del/4RK-CFTR) onto clear-bottom 384-well black microplates (2500 cells/well), 1 µg/mL doxycycline (#9891, Sigma-Aldrich, St. Louis, MO, USA) was added to cell culture medium to induce CFTR expression, and 24 h later, compounds were administered. After an additional 24 h, extracellular Flag tags were immunostained with an anti-Flag M2 monoclonal antibody (1:500, #F1804, Sigma-Aldrich) and an anti-mouse Alexa Fluor^®^ 647 conjugated secondary antibody (1:500, #A31571, Molecular Probes) using a protocol without cell permeabilization, as described [14,33]. The nuclei were counterstained with a Hoechst 33,342 solution (1:5000, #S2261, Sigma-Aldrich) and used for contrast-based autofocus. Cell imaging was performed in an inverted widefield microscope Leica DMI6000 (Leica Microsystems, Wetzlar, Germany) equipped with a 12-bit 1344 × 1024-pixel resolution DFC360FX camera and a 10× objective. Automatic image analysis was performed using a pipeline developed to measure CFTR trafficking efficiency [33]. The stained Flag tags enable quantification of the CFTR located exclusively at the PM, while mCherry tags report the total amount of CFTR protein expressed by each individual cell. Using this pipeline, the background was first subtracted from each image to correct for illumination and background fluorescence. Thereafter, a quality control was applied to remove cells that do not express CFTR, exhibit abnormal morphology, or contain a significant number of saturated pixels. Finally, total and PM CFTR were measured in each cell using the fluorescence quantification, as described [33]. All conditions were performed in triplicate in each plate.

### 2.5. HS-YFP Assay on the Plate Reader

CFBE cells expressing WT- or F508del-CFTR together with the HS-YFP (H148Q/I152L) were seeded (50,000 cells/well) onto clear-bottom 96-well black microplates. After 24 h, the cells were washed with phosphate-buffered saline (PBS) containing (in mM): 137 NaCl, 2.7 KCl, 8.1 Na_2_HPO_4_, 1.5 KH_2_PO_4_, 1.0 CaCl_2_, and 0.5 MgCl_2_. The cells were then incubated for 30 min with 60 µL of PBS plus Fsk (20 µM) and Gen (50 µM) to maximize stimulation of CFTR channels. Thereafter, the cells were transferred to a microplate reader (Tecan Infinite 200 Pro) for CFTR activity determination. The plate reader was equipped with high-quality excitation (485 ± 20 ηm) and emission (535 ± 25 ηm) filters for YFP. The assay consists of a continuous 14-sec fluorescence reading with 2-sec before and 12-sec after injection of an iodide (I^−^)-containing solution (PBS with Cl^−^ replaced by I^−^, final I^−^ concentration: 100 mM). Data were normalized to the initial background-subtracted fluorescence. To determine I^−^ influx rate, the final 11-sec of the data points for each well were fitted with an exponential function to extrapolate initial slope (*dF*/*dt*) [22,32]. All conditions were performed in triplicate in each plate.

### 2.6. Western Blot

Cells were lysed using a lysis buffer (31.25 mM Tris-HCl pH 6.8, 1.5% SDS [*w*/*v*], 5% glycerol and 0.5 mM DTT) supplemented with complete protease inhibitor cocktail (Roche, Basel, Switzerland) and Laemmli sample buffer (#1610747, Bio-Rad). Whole-cell lysates were then subjected to SDS-PAGE 10% gel (*w*/*v*) and transferred to a PVDF membrane (Millipore, MA, USA). CFTR was detected using the monoclonal anti-human CFTR antibody 596 (1:3000, from CF Foundation Therapeutics [CFFT]) and the blotting-grade horseradish peroxidase secondary antibody (1:3000, Bio-Rad). Anti-calnexin (1:3000; BD Biosciences) or anti-tubulin antibodies (1:10,000; Sigma-Aldrich) were used as a loading control. Proteins were detected using the antibodies mentioned above and subsequently visualized by chemiluminescence using the Clarity Western ECL substrate (Bio-Rad) in a Chemidoc XRS system. Bands were analyzed with Image™ Lab software version 6.0 (Bio-Rad).

### 2.7. FLIPR Membrane Potential (FMP) Assay

FRT cells expressing CFTR variants (G85E, R334W, T338I, R347P, F508del, V520F, S549R, M1101K, and N1303K) were seeded (25,000 cells/well) onto 96-well plates. After 24 h, cells were incubated with compounds, washed with PBS, then exposed to a solution containing the voltage sensitive FLIPR fluorescent dye and incubated at room temperature for 5 min as described [29]. Gen (50 µM) was added to media and gently mixed, and the plate was placed in a plate reader to read fluorescence. Fsk (10 µM) was then added and the increase in signal was used to calculate the mean initial rate as a measure of CFTR function.

### 2.8. Fsk-Induced Swelling (FIS) Assay of Intestinal Organoids

Crypt isolation from rectal biopsies, culture of organoids (F508del/F508del genotype), and FIS assay was performed as described [27]. Twenty-four hours after seeding and treatment with MCG1516A and VX-661, organoids were acutely stimulated (30 min) with Fsk (0.02–5 µM) plus VX-770 (3 µM) and live-cell imaging was performed using a confocal microscopy (Leica TCS SP8) with a 5× objective for 60 min at 37 °C. FIS was quantified using the area under the curve (AUC, *t* = 60 min, baseline = 100%) and a CellProfiler-based algorithm.

### 2.9. Statistical Analyses 

All conditions were performed in at least three independent experiments. Statistical comparisons were performed using one-way ANOVA followed by Dunnett’s or Tukey’s post hoc tests in the GraphPad Prism software version 8.0.2 (GraphPad, San Diego, CA, USA). Differences were considered statistically significant when *p* value was less than 0.05.

## 3. Results

### 3.1. Assessment of MCG1516A Effects on F508del-CFTR Processing, PM Expression and Channel Activity and Its Additive Effects to Those of the FDA-Approved Corrector VX-661

**Figure 1:**
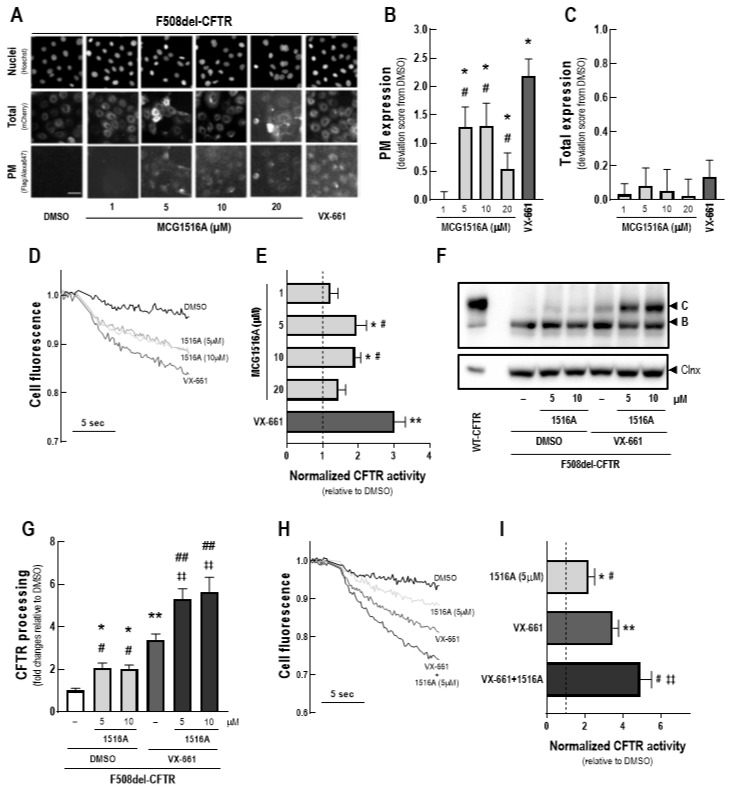
**Assessment of MCG1516A effects on F508del-CFTR processing, trafficking to the PM and channel activity and its additive effects in combination with the FDA-approved corrector VX-661**. (**A**) Representative fluorescence images of CFBE cells expressing mCherry-Flag-F508del-CFTR incubated with DMSO (vehicle), increasing concentration of MCG1516A (1 to 20 µM) or VX-661 (5 µM) for 24 h. Scale bar = 50 µm. The extracellularly exposed Flag-tags and mCherry-tags were quantified to determine (**B**) CFTR located at the PM and (**C**) the total expression of CFTR in cells. Data are represented as deviation score (+SD) relative to the negative control (DMSO), *n* = 4. Statistical analysis was performed using One-way ANOVA followed by Tukey’s post hoc test: vs. DMSO: * *p* < 0.05; vs. VX-661: ^#^ *p* < 0.05. (**D**) Representative cell fluorescence recording acquired with a microplate reader. CFBE cells co-expressing F508del-CFTR and the HS-YFP were incubated for 24 h with DMSO, MCG1516A (1 to 20 µM), or VX-661 (5 µM). Cells were then stimulated for 30 min with Fsk (20 µM) plus Gen (50 µM). (**E**) CFTR activity was quantified based on the rate of YFP quenching and normalized to the negative control (DMSO, dashed line). Data are represented as means + SD, *n* = 4. Statistical analysis was performed using One-way ANOVA followed by Tukey’s post hoc test: vs. DMSO: * *p* < 0.05, ** *p* < 0.01; vs. VX-661: ^#^ *p* < 0.05. (**F**) Representative immunoblotting images of CFBE cells expressing WT- or F508del-CFTR incubated individually or in combination with DMSO (vehicle), MCG1516A (5 or 10 µM), and VX-661 (5 µM) for 24 h. (**G**) CFTR processing [C/(B + C)] was quantified and normalized to calnexin (Clnx) levels (loading control). Data are represented as means + SD, *n* = 4. Statistical analysis was performed using One-way ANOVA followed by Tukey’s post hoc test: vs. DMSO: * *p* < 0.05, ** *p* < 0.01; vs. VX-661: ^#^ *p* < 0.05, ^##^ *p* < 0.01; vs. 1516A (5 µM): ^‡‡^ *p* < 0.01. (**H**) Representative cell fluorescence recording of CFBE cells co-expressing F508del-CFTR and the HS-YFP incubated for 24 h with DMSO, MCG1516A (5 µM), and VX-661 (5 µM) individually or in combination. Cells were then stimulated for 30 min with Fsk (20 µM) plus Gen (50 µM). (**I**) CFTR activity was quantified based on the rate of YFP quenching and normalized to the negative control (DMSO, dashed line). Data are represented as means + SD, *n* = 4. Statistical analysis was performed using One-way ANOVA followed by Tukey’s post hoc test: vs. DMSO: * *p* < 0.05, ** *p* < 0.01; vs. VX-661: ^#^ *p* < 0.05, vs. 1516A (5 µM): ^‡‡^ *p* < 0.01.

To assess the effects of MCG1516A on F508del-CFTR total expression and PM trafficking, we first performed immunofluorescence detection of the mCherry-tag and Flag-tag of mCherry-Flag-F508del-CFTR expressed in CFBE cells without cell permeabilization (Figure 1A). After 24 h, treatment with MCG1516A resulted in the appearance of the anti-Flag signal, indicating that F508del-CFTR PM expression was rescued, and the maximal signal was achieved at concentrations 5 and 10 µM, although this effect was lower to that of VX-661 (Figure 1B). Neither MCG1516A, at different concentrations (1–20 µM), nor VX-661 induced alterations in the total amount of CFTR, as the mCherry fluorescence signal was similar of that for DMSO (negative control) (Figure 1C).

Next, the ability of MCG1516A to rescue F508del-CFTR activity was evaluated by measuring the HS-YFP quenching rate promoted by I^−^ influx into CFBE cells expressing F508del-CFTR together with the HS-YFP (Figure 1D). After 24 h, both MCG1516A (5 and 10 µM) and VX-661 rescued F508del-CFTR activity upon Fsk + Gen stimulation, although the efficacy of MCG1516A was lower compared to that of VX-661 (Figure 1E).

Rescue of F508del-CFTR processing by MCG1516A was further confirmed by WB through the appearance of the fully-glycosylated mature form of CFTR (~180 kDa, band C), while DMSO only led to the appearance of the core-glycosylated immature form of CFTR (~140 kDa, band B) (Figure 1F,G). VX-661 also rescued F508del-CFTR processing at higher efficacy when compared to MCG1516A. An even higher rescue of F508del-CFTR processing was obtained when cells were treated with MCG1516A and VX-661 together compared to each individually. Such results were confirmed by the HS-YFP quenching rate in which a higher rescue of F508del-CFTR activity was obtained by the co-treatment with MCG1516A and VX-661 compared to their individual effects (Figure 1H,I).

### 3.2. Assessment of Rescue of CFTR-Dependent Swelling in F508del/F508del Intestinal Organoids by MCG1516A

**Figure 2:**
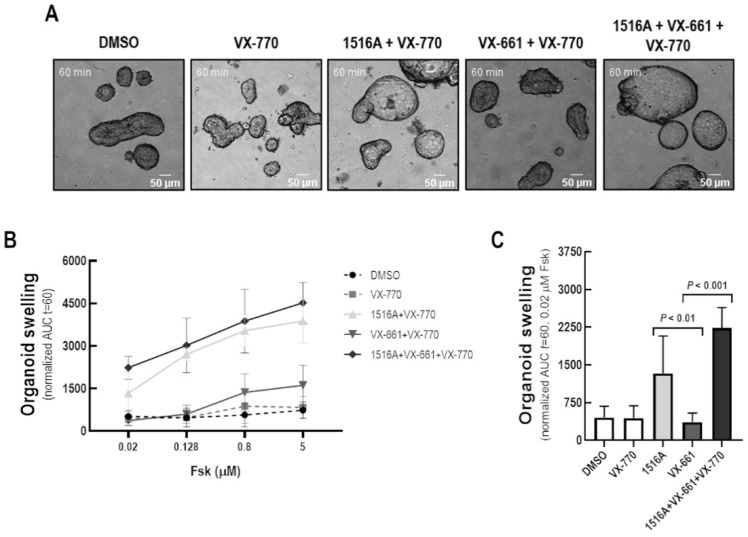
**Assessment of rescue of CFTR-dependent fluid secretion in F508del/F508del intestinal organoids by MCG1516A.** (**A**) Images of F508del/F508del intestinal organoids (ID: CFL76) incubated for 24 h with DMSO (negative control), MCG1516A (5 µM), and/or VX-661 (5 µM) and acutely stimulated (30 min) with Fsk (0.02 µM) at different concentrations (0.02 to 5 µM) plus VX-770 (1 µM). (**B**,**C**) Quantification of FIS of organoids (baseline = 100%, *t* = 60 min). Data are represented as means + SD, *n* = 3. Statistical analysis was calculated by One-way ANOVA using Fischer’s LSD test.

The ability of MCG1516A to rescue F508del-CFTR activity was further evaluated using the FIS assay of intestinal organoids derived from CF individuals with the F508del/F508del genotype (Figure 2). After 24 h-treatment with MCG1516A, organoid FIS values were higher than those obtained for VX-661, upon Fsk + VX-770 stimulation. Even higher FIS values were observed for organoids treated for 24 h with both MCG1516A and VX-661 than their effects individually.

### 3.3. Assessment of MCG1516A Effects on WT-CFTR Processing, PM Expression and Channel Activity and Its Additive Effects with the FDA-Approved Corrector VX-661

**Figure 3:**
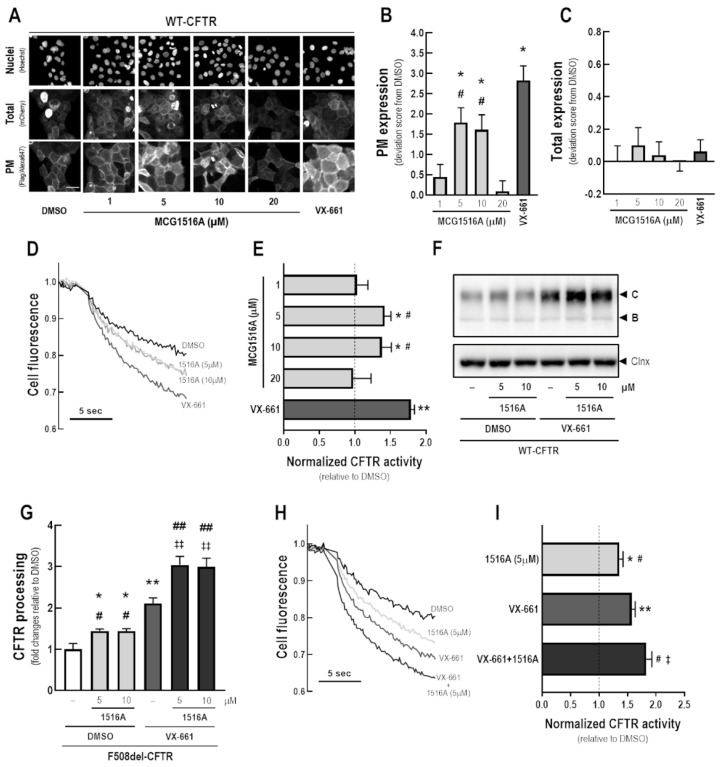
**Assessment of MCG1516A effects on WT-CFTR processing, PM expression and channel activity and its additive effects in combination with the FDA-approved corrector VX-661.** (**A**) Representative fluorescence images of CFBE cells expressing mCherry-Flag-WT-CFTR incubated with DMSO (vehicle), increasing concentration of MCG1516A (1 to 20 µM) or VX-661 (5 µM) for 24 h. Scale bar = 50 µm. The extracellularly exposed Flag-tags and mCherry-tags were quantified to determine (**B**) CFTR located at the PM and (**C**) the total expression of CFTR in cells. Data are represented as deviation score (+SD) relative to the negative control (DMSO), *n* = 4. Statistical analysis was performed using One-way ANOVA followed by Tukey’s post hoc test: vs. DMSO: * *p* < 0.05; vs. VX-661: ^#^ *p* < 0.05. (**D**) Representative cell fluorescence recording acquired with a microplate reader. CFBE cells co-expressing WT-CFTR and the HS-YFP were incubated for 24 h with DMSO, MCG1516A (1 to 20 µM), or VX-661 (5 µM). Cells were then stimulated for 30 min with Fsk (2 µM) plus Gen (50 µM). (**E**) CFTR activity was quantified based on the rate of YFP quenching and normalized to the negative control (DMSO, dashed line). Data are represented as means + SD, *n* = 4. Statistical analysis was performed using One-way ANOVA followed by Tukey’s post hoc test: vs. DMSO: * *p* < 0.05, ** *p* < 0.01; vs. VX-661: ^#^ *p* < 0.05. (**F**) Representative immunoblotting images of CFBE cells expressing WT-CFTR incubated individually or in combination with DMSO (vehicle), MCG1516A (5 or 10 µM), and VX-661 (5 µM) for 24 h. (**G**) CFTR processing [C/(B + C)] was quantified and normalized to calnexin (Clnx) levels (loading control). Data are represented as means + SD, *n* = 4. Statistical analysis was performed using One-way ANOVA followed by Tukey’s post hoc test: vs. DMSO: * *p* < 0.05, ** *p* < 0.01; vs. VX-661: ^#^ *p* < 0.05, ^##^ *p* < 0.01; vs. 1516A (5 µM): ^‡‡^ *p* < 0.01. (**H**) Representative cell fluorescence recording of CFBE cells co-expressing WT-CFTR and the HS-YFP incubated for 24 h with DMSO, MCG1516A (5 µM), and VX-661 (5 µM) individually or in combination. Cells were then stimulated for 30 min with Fsk (2 µM) plus Gen (50 µM). (**I**) CFTR activity was quantified based on the rate of YFP quenching and normalized to the negative control (DMSO, dashed line). Data are represented as means + SD, *n* = 4. Statistical analysis was performed using One-way ANOVA followed by Tukey’s post hoc test: vs. DMSO: * *p* < 0.05, ** *p* < 0.01; vs. VX-661: ^#^ *p* < 0.05, vs. 1516A (5 µM): ^‡^ *p* < 0.05.

After 24 h-treatment of CFBE cells expressing mCherry-Flag-WT-CFTR with MCG1516A at concentrations 5 and 10 µM, an increase in WT-CFTR PM expression was observed, as evidenced by an increase in the anti-Flag signal, although at lower efficacy than for VX-661 (Figure 3A,B). Cells treated with DMSO, MCG1516A (1 to 20 µM), or VX-661 demonstrated a comparable mCherry fluorescence signal, indicating that there were no differences in the total amount of CFTR (Figure 3C).

To assess the effects of MCG1516A on WT-CFTR activity, CFBE cells co-expressing WT-CFTR and the HS-YFP were treated with compounds for 24 h and the HS-YFP quenching rate promoted by I^−^ influx was measured. Upon Fsk + Gen stimulation, both MCG1516A (5 and 10 µM) and VX-661 demonstrated an increase in WT-CFTR activity, with VX-661 having a larger effect than MCG1516A (Figure 3D,E).

WT-CFTR processing was increased in CFBE cells treated with MCG1516A (5 and 10 µM) or VX-661 compared to DMSO (Figure 3F,G). Although VX-661 was more effective than MCG1516A, co-treatment with both correctors had even greater effects. Similar results were obtained by measuring the HS-YFP quenching rate, in which WT-CFTR activity was further increased by the co-treatment with MCG1516A and VX-661 compared to the effect of each compound individually (Figure 3H,I).

### 3.4. Assessment of Additive Effects of MCG1516A to Low Temperature in Rescuing F508del-CFTR Processing and Activity 

**Figure 4:**
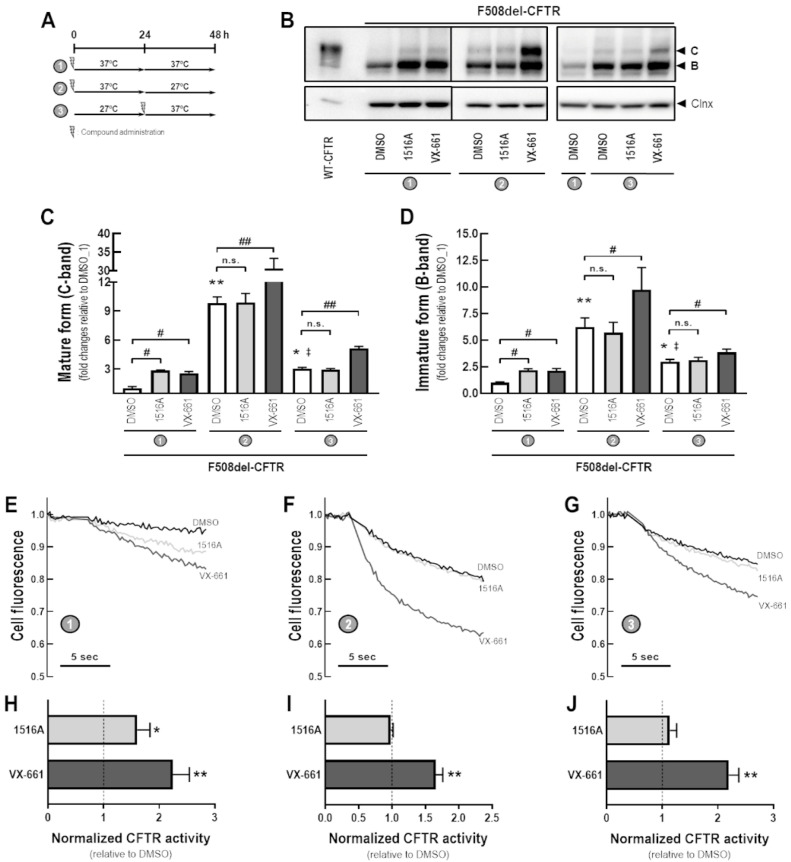
**Assessment of the additive effects of MCG1516A to a low temperature in rescuing F508del-CFTR processing and activity.** (**A**) Schematic flow chart and timeline of experiments: (1) cells were incubated with compounds for 48 h at 37 °C; (2) cells were incubated with compounds for 24 h at 37 °C and then culture for an additional 24 h at 27 °C; (3) cells were cultured for 24 h at 27 °C and then compounds were administered and cells were cultured for an additional 24 h at 37 °C. (**B**) Representative immunoblotting images of CFBE cells expressing WT- or F508del-CFTR incubated with DMSO (vehicle), MCG1516A (5 µM), or VX-661 (5 µM). (**C**,**D**) The mature and immature forms of CFTR (C-band and B-band, respectively) were quantified and normalized to calnexin (Clnx) levels (loading control). Data are represented as means + SD, *n* = 4. Statistical analysis was performed using One-way ANOVA followed by Tukey’s post hoc test: vs. DMSO_1: * *p* < 0.05, ** *p* < 0.01; vs. DMSO_2: ^‡^ *p* < 0.05; Compound vs. respective DMSO (white bars): ^#^ *p* < 0.01, ^##^ *p* < 0.01; n.s.: not significant. (**E**–**G**) Representative cell fluorescence recording of CFBE cells co-expressing F508del-CFTR and the HS-YFP incubated with DMSO, MCG1516A (5 µM), and VX-661 (5 µM). Cells were then stimulated for 30 min with Fsk (20 µM) plus Gen (50 µM). (**H**–**J**) CFTR activity was quantified based on the rate of YFP quenching and normalized to the negative control (DMSO, dashed line). Data are represented as means + SD, *n* = 4. Statistical analysis was performed using One-way ANOVA followed by Tukey’s post hoc test: vs. DMSO: * *p* < 0.05, ** *p* < 0.01.

To better understand the MoA by which MCG1516A rescues F508del-CFTR processing and activity, we evaluated its effects under low temperature incubation (27 °C) for 24 h prior to or after the presence of MCG1516A and VX-661 for an additional 24 h at 37 °C (Figure 4A). After 48 h at 37 °C, CFBE cells expressing F508del-CFTR that had been treated with MCG1516A or VX-661 had increased levels of mature and immature CFTR glycoforms compared to DMSO controls (Figure 4B–D). Cells treated with DMSO at 37 °C for 24 h and then switched to 27 °C for an additional 24 h had elevated levels of both mature and immature CFTR compared to cells maintained at 27 °C for 24 h and then switched to 37 °C for an additional 24 h or maintained at 37 °C for 48 h. In cells maintained at 37 °C for 24 h and then at 27 °C for an additional 24 h, VX-661, but not MCG1516, significantly increased the amount of mature and immature forms of CFTR compared to DMSO. A similar effect was observed in cells maintained at 27 °C for 24 h and then at 37 °C for an additional 24 h.

The same approach was employed in CFBE cells co-expressing F508del-CFTR and the HS-YFP to evaluate the rescue of F508del-CFTR activity by measuring the HS-YFP quenching rate (Figure 4E–J). Compared to DMSO, both MCG1516A and VX-661 rescued F508del-CFTR responses to Fsk+Gen stimulation when cells were maintained at 37 °C for 48 h, although MCG1516A was less effective than VX-661. In cells maintained at 37 °C for 24 h and then at 27 °C for an additional 24 h, MCG1516A did not further enhance functional rescue of F508del-CFTR, whereas VX-661 did cause a significant increase, and the same behavior was observed for cells maintained at 27 °C for 24 h and then at 37 °C for additional 24 h.

### 3.5. Assessment of Potentiator Activity of MCG1516A on the Rescued F508del-CFTR

**Figure 5:**
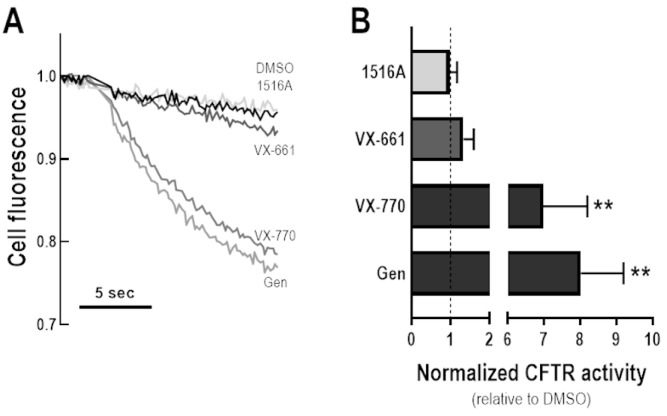
**Assessment of potentiator activity of MCG1516A on the rescued F508del-CFTR.** (**A**) Representative cell fluorescence recording of CFBE cells co-expressing F508del-CFTR and the HS-YFP incubated for 24 h at 27 °C. Cells were then stimulated for 30 min with Fsk (20 µM) plus DMSO (vehicle), MCG1516A (5 µM), VX-661 (5 µM), VX-770 (1 µM), or Gen (50 µM). (**B**) CFTR activity was quantified based on the rate of YFP quenching and normalized to the negative control (DMSO, dashed line). Data are represented as means + SD, *n* = 4. Statistical analysis was performed using One-way ANOVA followed by Tukey’s post hoc test: vs. DMSO: ** *p* < 0.01.

To investigate whether MCG1516A might also act as a potentiator, CFBE cells co-expressing F508del-CFTR and the HS-YFP were incubated at 27 °C for 24 h, then acutely stimulated (30 min) with Fsk plus MCG1516A, VX-661, DMSO (negative control), Gen, or VX-770 (positive controls), and the HS-YFP quenching rate was measured (Figure 5). In cells incubated at a low temperature, there was a significant decay in cell fluorescence upon stimulation with either Fsk + Gen or Fsk + VX-770, indicating I^−^ influx through rescued F508del-CFTR. On the other hand, acute stimulation with Fsk + MCG1516A or Fsk + VX-661 yielded cell fluorescence comparable to Fsk + DMSO. These results indicate that a gating defect persists after F508del-CFTR has been partially rescued by low temperature, and that defect was not corrected by either of these compounds.

### 3.6. Assessment of Additive Effects of MCG1516A to a Low Temperature in Enhancing WT-CFTR Processing and Activity

**Figure 6:**
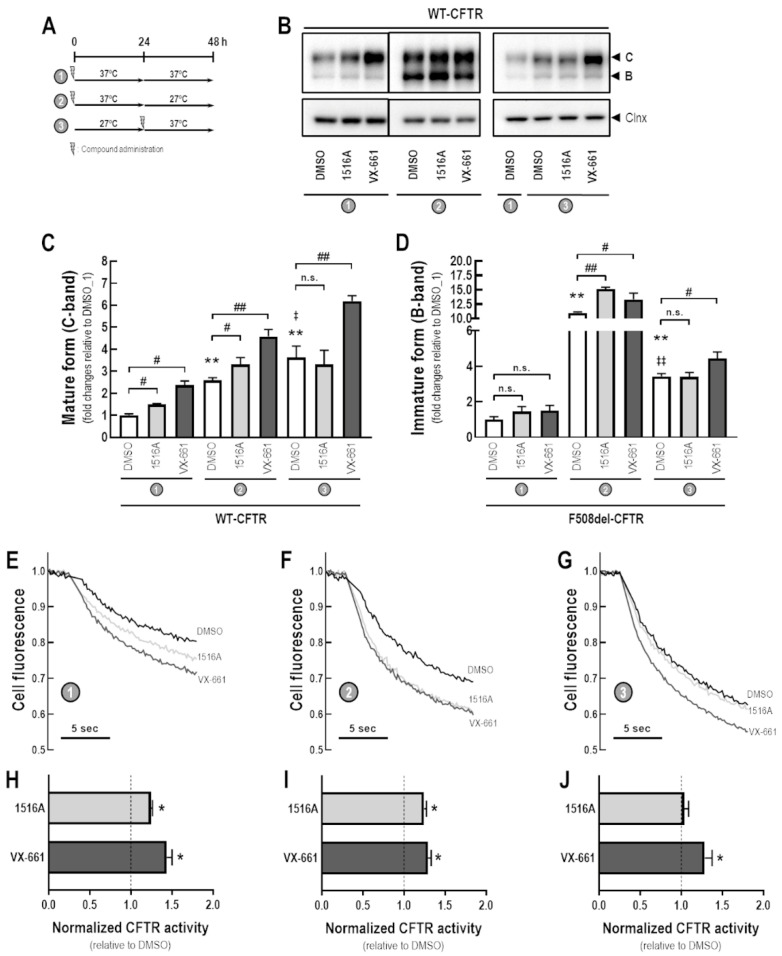
**Assessment of additive effects of MCG1516A to a low temperature in enhancing WT-CFTR processing and activity.** (**A**) Schematic flow chart and timeline of experiments: (1) cells were incubated with compounds for 48 h at 37 °C; (2) cells were incubated with compounds for 24 h at 37 °C and then culture for an additional 24 h at 27 °C; (3) cells were cultured for 24 h at 27 °C and then compounds were administered and cells were cultured for an additional 24 h at 37 °C. (**B**) Representative immunoblotting images of CFBE cells expressing WT-CFTR incubated with DMSO (vehicle), MCG1516A (5 µM), or VX-661 (5 µM). (**C**,**D**) The mature and immature forms of CFTR (C-band and B-band, respectively) were quantified and normalized to calnexin (Clnx) levels (loading control). Data are represented as means + SD, *n* = 4. Statistical analysis was performed using One-way ANOVA followed by Tukey’s post hoc test: vs. DMSO_1: ** *p* < 0.01; vs. DMSO_2: ^‡^ *p* < 0.05, ^‡‡^ *p* < 0.01; Compound vs. respective DMSO (white bars): ^#^ *p* < 0.05, ^##^ *p* < 0.01; n.s.: not significant. (**E**–**G**) Representative cell fluorescence recording of CFBE cells co-expressing WT-CFTR and the HS-YFP incubated with DMSO, MCG1516A (5 µM), and VX-661 (5 µM). Cells were then stimulated for 30 min with Fsk (2 µM) plus Gen (50 µM). (**H**–**J**) CFTR activity was quantified based on the rate of YFP quenching and normalized to the negative control (DMSO, dashed line). Data are represented as means + SD, *n* = 4. Statistical analysis was performed using One-way ANOVA followed by Tukey’s post hoc test: vs. DMSO: * *p* < 0.05.

Next, we evaluated the potential additive effects of a low temperature and MCG1516A on WT-CFTR processing and activity (Figure 6A). After 48 h at 37 °C, both MCG1516A and VX-661 increased the amount of mature CFTR, but not immature form, when compared with DMSO controls (Figure 6B–D). Cells maintained at 27 °C for 24 h and then treated with DMSO and maintained at 37 °C for an additional 24 h had elevated levels of both the mature and immature forms of CFTR compared to cells maintained at 37 °C (24 h) → 27 °C (24 h) or maintained at 37 °C for 48 h. MCG1516A and VX-661 increased total CFTR (both mature and immature forms) compared to DMSO when cells were maintained at 37 °C (24 h) → 27 °C (24 h). When cells were maintained at 27 °C for 24 h and then at 37 °C for additional 24 h, VX-661, but not MCG1516A, increased the amount of both mature and immature forms of CFTR compared to DMSO. Similar behavior was observed for the assessment of WT-CFTR activity by measuring the HS-YFP quenching rate in CFBE cells co-expressing WT-CFTR and the HS-YFP (Figure 6E–J).

### 3.7. Assessment of the Effects of MCG1516A on Processing and PM Expression of F508del-CFTR Genetic Revertants and the Traffic-Null Variant DD/AA 

**Figure 7:**
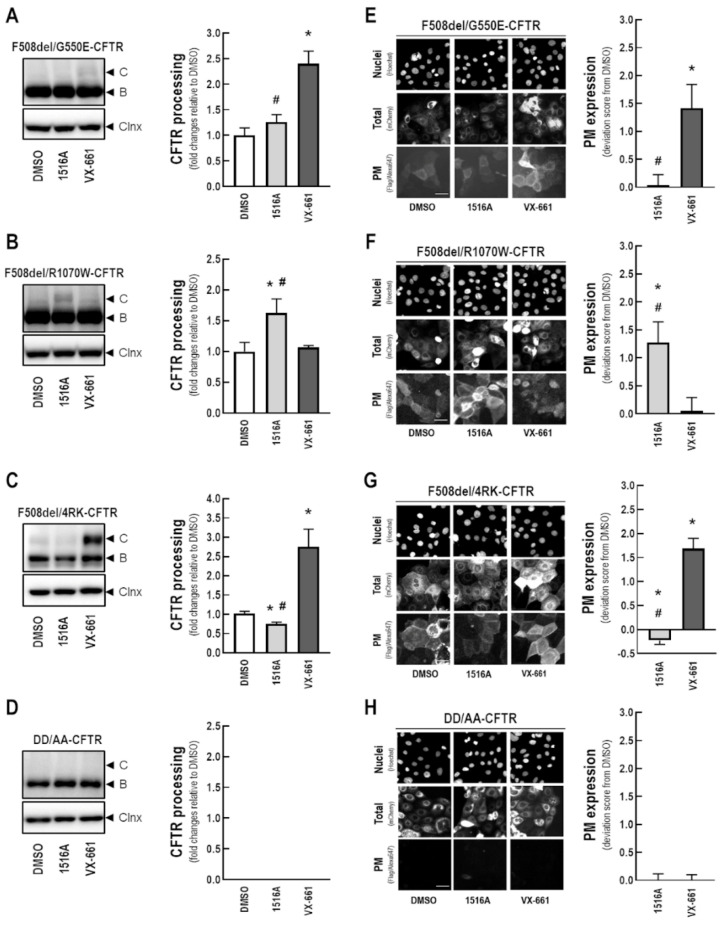
**Assessment of MCG1516A effects on processing and PM expression of F508del-CFTR genetic revertants or the traffic-null variant DD/AA**. (**A**–**D**) Representative immunoblotting images of CFBE cells expressing (**A**) F508del/G550E-, (**B**) F508del/R1070W-, (**C**) F508del/4RK-, and (**D**) DD/AA-CFTR incubated with DMSO (vehicle), MCG1516A (5 µM), or VX-661 (5 µM) for 24 h. CFTR processing [C/(B + C)] was quantified and normalized to calnexin (Clnx) levels (loading control). Data are represented as means + SD, *n* = 4. Statistical analysis was performed using One-way ANOVA followed by Tukey’s post hoc test: vs. DMSO: * *p* < 0.05; vs. VX-661: ^#^ *p* < 0.05. (**E**–**H**) Representative fluorescence images of CFBE cells expressing mCherry-Flag-CFTR variants [(**E**) F508del/G550E-, (**F**) F508del/R1070W-, (**G**) F508del/4RK-, and (**H**) DD/AA-CFTR] incubated with DMSO (vehicle), MCG1516A (5 µM), or VX-661 (5 µM) for 24 h. Scale bar = 50 µm. The extracellularly exposed Flag-tags were quantified to determine CFTR PM expression. Data are represented as deviation score (+SD) relative to the negative control (DMSO), *n* = 4. Statistical analysis was performed using One-way ANOVA followed by Tukey’s post hoc test: vs. DMSO: * *p* < 0.05; vs. VX-661: ^#^ *p* < 0.05.

To shed further light on the MoA of MCG1516A in rescuing F508del-CFTR, we evaluated its additive effects on the processing and PM expression of genetic revertants of this mutant. For this purpose, CFBE cells expressing either F508del-CFTR or mCherry-Flag-F508del-CFTR in *cis* with the revertants G550E (Figure 7A,E), R1070W (Figure 7B,F), and 4RK (Figure 7C,G) were treated with MCG1516A or VX-661 for 24 h. In parallel, CFBE cells expressing the traffic-null variant DD/AA (either with or without double tag) were also treated with these compounds for 24 h (Figure 7D,H). F508del/G550E-CFTR processing and PM expression were increased in cells treated with VX-661, but not with MCG1516A (Figure 7A,E). On the other hand, treatment with MCG1516A, but not VX-661, increased F508del/R1070W-CFTR processing and PM expression (Figure 7B,F). Such findings suggest that these compounds rescue F508del-CFTR processing and PM trafficking by distinct mechanisms. In cells expressing F508del/4RK-CFTR, VX-661 further increases its processing and PM expression, while MCG1516A significantly reduced F508del/4RK-CFTR processing and PM expression compared to DMSO (Figure 7C,G). Neither MCG1516A, nor VX-661, were able to rescue DD/AA-CFTR processing and PM expression (Figure 7D,H).

### 3.8. Assessment of MCG1516A Effects on Other CF-Causing Mutations 

**Figure 8:**
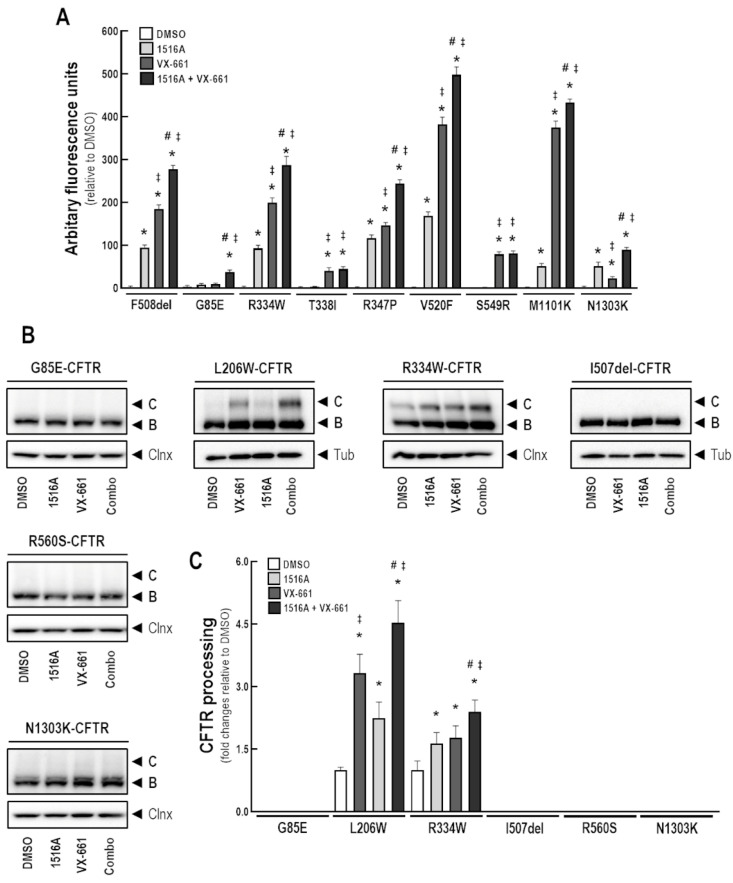
**Assessment of MCG1516A effects on other CF-causing mutations.** (**A**) FRT cells constitutively expressing CFTR mutations (F508del, G85E, R334W, T338I, R347P, V520F, S549R, M1101K, and N1303K) were incubated for 24 h with DMSO (negative control), MCG1516A (5 µM), and VX-661 (5 µM), individually or in combination. FMP assay was performed to determine membrane depolarization promoted by stimulation with Fsk (10 µM) plus Gen (50 µM) as a readout of CFTR function. Data are represented as means + SD, *n* = 4. Statistical analysis was performed using One-way ANOVA followed by Tukey’s post hoc test: vs. DMSO: * *p* < 0.05; vs. 1516A: ^‡^ *p* < 0.05; vs. VX-661: ^#^ *p* < 0.05. (**B**) Representative immunoblotting images of CFBE cells expressing the CFTR mutants (G85E, L206W, R334W, I507del, R560S, or N1303K) incubated with DMSO (vehicle), MCG1516A (5 µM), and/or VX-661 (5 µM) for 24 h. (**C**) CFTR processing [C/(B + C)] was quantified and normalized to loading control levels (calnexin [Clnx] or tubulin [Tub]). Data are represented as means + SD, *n* = 3–4. Statistical analysis was performed using One-way ANOVA followed by Tukey’s post hoc test: vs. DMSO: * *p* < 0.05; vs. 1516A: ^‡^ *p* < 0.05; vs. VX-661: ^#^ *p* < 0.05.

To assess the effects of MCG1516A in rescuing other CFTR mutants, we initially performed the FMP assay in FRT cells constitutively expressing G85E-, R334W-, T338I-, R347P-, V529F-, S549R-, M1101K-, and N1303K-CFTR (in parallel with F508del-CFTR as a positive control) to measure the depolarization that occurs once CFTR PM channels are activated. Cells were treated with MCG1516A and VX-661 individually, or combined, for 24 h (Figure 8A). Upon Fsk+Gen stimulation, an increase response was found in FRT cells expressing F508del-CFTR treated with either MCG1516A or VX-661, as expected, and a higher effect was observed when these compounds were combined, consistent with the above data in CFBE cells. FRT cells expressing R334W-, R347P-, V520F-, and M1101K-CFTR demonstrated a similar behavior of F508del-expressing cells, by displaying an increase in the Fsk+Gen response after treatment with MCG1516A or VX-661, although the latter was more effective than the former. Co-treatment with both compounds promoted an even greater response in these cells. On the other hand, T338I- and S549R-expressing cells only responded to VX-661 treatment with no additive effects when MCG1516A was co-administered. In this assay, G85E- and N1303K-expressing cells also demonstrated a response to treatments, although at very low levels compared to other mutants. An effect on G85E-expressing cells was demonstrated only when MCG1516A and VX-661 were used together, while N1303K-expressing cells responded to both MCG1516A and VX-661 individually, with additive effect when these were used together.

We further evaluated the effects of MCG1516A and VX-661 on CFBE cells expressing G85E-, L206W-, R334W-, I507del-, R560S-, and N1303K-CFTR (Figure 8B,C). Cells were treated for 24 h with these compounds individually or combined and whole-cell lysates were subjected to WB analysis. Treatment with MCG1516A or VX-661 resulted in the appearance and increased amount, respectively, of the fully-glycosylated mature form of CFTR in L206W- and R334W-expressing cells. Such effects were even greater when these compounds were used together. However, in G85E-, I507del-, R560S-, and N1303K-expressing cells, neither MCG1516A nor VX-661, alone or combined, were able to rescue CFTR processing, thus, only the presence of the core-glycosylated immature form of CFTR was detected, similarly to DMSO.

## 4. Discussion

This study aimed to characterize the effects of corrector MCG1516A both in cell lines constitutively expressing WT-, F508del-CFTR, and several other CF-causing mutations and in intestinal organoids (F508del/F508del genotype). We also shed light on the MoA of MCG1516A by examining its additivity to FDA-approved corrector VX-661, low temperature and genetic revertants of F508del-CFTR.

The development of correctors VX-661 and VX-445 constituted a landmark in the medicinal perspective for most individuals with CF. Indeed, the ‘highly effective’ triple combination of CFTR modulators, which is composed of these two correctors plus the potentiator VX-770, has demonstrated substantial clinical benefit for individuals with CF carrying the F508del mutation in at least one allele [19,20]. More recently, the FDA has extended the approval of this combined therapy to 177 additional CF-causing mutations based on in vitro results in FRT cells, so far without formal publication of these data [16]. Despite such progress, real-life and long-term effects of this combined therapy have yet to be demonstrated. Furthermore, the triple combination VX-445/VX661/VX-770 only partially rescues F508del-CFTR stability, trafficking, and function [22], suggesting that further improvements can be made. Efficacy may also differ among different mutations, namely for those in the same functional class, and some CFTR mutations demonstrated to be only modestly corrected (i.e., below therapeutically relevant levels) or not rescued at all by this combined therapy [25]. Accordingly, novel corrector drugs (possibly as combinations) are still needed to provide more robust therapeutic effects.

The MoA of these drugs remains poorly elucidated, and a better understanding of these mechanisms may provide new pathways for the development of more effective therapies. Here, we took a closer look at the MoA of MCG1516A, a newly developed F508del-CFTR corrector [29]. Our immunofluorescence and biochemical results demonstrated that MCG1516A rescues F508del-CFTR processing and trafficking to the PM in CFBE cells, albeit at lower levels than those of VX-661 (Figure 1). These data are consistent with a previous report demonstrating the efficacy of this compound in baby hamster kidney (BHK) cells constitutively expressing F508del-CFTR [29]. As we found no changes in the total amount of F508del-CFTR, the rescue by MCG1516A and VX-661 is unlikely to be a consequence of an increase in CFTR protein synthesis. MCG1516A was also able to restore F508del-CFTR-dependent Cl^−^ secretion in polarized human bronchial epithelial (HBE) cells (F508del/F508del) [29]. This is in agreement with our findings demonstrating the rescue of F508del-CFTR function by this compound in different assays, namely the HS-YFP assay on a plate reader in CFBE cells (Figure 1) and by the FMP assay in FRT cells (Figure 8). Because the use of samples from individuals with CF has become an important tool to predict clinical effectiveness [2,3], we further validated our results in intestinal organoids obtained from an individual with CF (F508del/F508del genotype). Surprisingly, data from the FIS assay indicated that MCG1516A rescues F508del-CFTR function at higher efficacy than VX-661 (Figure 2). Such difference in efficacy may be attributed to the use of different cell systems, since previous reports demonstrated that the cell background and polarization state impact, not only on CFTR processing, but also on corrector efficacy [8,31,32,34]. Nevertheless, the co-treatment with MCG1516A and VX-661 demonstrated additive effects in rescuing F508del-CFTR in all assays and cell models used herein (refer to Figure 1, Figure 2 and Figure 8), indicating that these compounds are likely to act by distinct MoA.

Because it is unknown whether MCG1516A may affect WT-CFTR processing, PM expression and function, we also assessed this in CFBE cells. Although WT-CFTR is a functional protein that reaches the PM, its biogenesis was described to be rather inefficient (25–30%) when overexpressed in some heterologous cell lines [35,36]. MCG1516A and VX-661 were able to increase both WT-CFTR processing and PM expression, as evidenced by our biochemical and immunofluorescence data (Figure 3). Furthermore, the increase in the HS-YFP quenching rate confirmed that both compounds enhance WT-CFTR function, most likely due to a higher number of WT-CFTR channels at the PM. In support of their distinct MoAs, greater effects were observed when WT-CFTR-expressing cells were co-treated with MCG1516A and VX-661, similarly to our findings for F508del-CFTR.

Processing of both WT- and F508del-CFTR is temperature sensitive and shifting cells from 37 °C to a more “permissive” state at 27 °C results in an enhancement of their folding and trafficking to the PM [7,8]. Our data confirmed that low temperature leads to accumulation of both core- and fully-glycosylated forms of F508del-CFTR, as well as rescued channel function (upon Fsk + Gen stimulation), but no additivity was observed in the presence of MCG1516A (Figure 4). A similar behavior was previously reported by the use of ouabain [9], indicating that these compounds and low temperature may share a similar mechanism. On the other hand, the rescue efficacy of VX-661 was significantly increased when in combination with low temperature, as also reported to correctors VRT-325, Corr4a, VX-809, and RDR01752 [13,14,37]. A higher rescue efficacy was observed when F508del-expressing cells were treated with VX-661 at 37 °C and then shifted to a lower temperature, possibly by promoting stabilization of the mutant protein followed by slowing down degradation, as previously proposed [13]. Despite the ability of MCG1516A and VX-661 to increase F508del-CFTR folding and trafficking, a certain amount of the fully-glycosylated form of F508del-CFTR was likely unstable and removed by peripheral quality control mechanisms when cells kept at 27 °C are returned to 37 °C, even in the presence of these compounds. Such effects also resulted in a lower quenching rate of the HS-YFP. These observations are consistent with the fact that, although the F508del-CFTR protein accumulates and reaches the PM at 27 °C, it can be reverted to a misfolded stage when cells are returned to 37 °C, being, thus, ubiquitinated and, subsequently, degraded [8,38,39]. Notably, the clinically approved correctors (VX-809, VX-661, and VX-445), alone or combined, were similarly not able to rescue F508del-CFTR protein stability to WT-CFTR levels [13,22,37]. Furthermore, MCG1516A and VX-661 were unable to restore channel gating when acutely administered, demonstrating the need to combine them with a potentiator to rescue channel gating (Figure 5). Altogether, these data suggest that MCG1516A and VX-661 should be combined with a stabilizer and a potentiator to maximize the rescue of F508del-CFTR.

Regarding the effects on WT-CFTR, both MCG1516A and VX-661 were additive to low temperature when cells were treated with compounds at 37 °C and then shifted to 27 °C, resulting in the accumulation of both fully- and core-glycosylated forms of CFTR and increased quenching rate of the HS-YFP (Figure 6). Such behavior was also reported previously for VX-809 and RDR01752 in terms of WT-CFTR PM expression [14]. In contrast, when compared to DMSO or MCG1516A, VX-661 demonstrated greater effects in shifting the accumulated core-glycosylated form of WT-CFTR to the fully-glycosylated one, when cells returned to 37 °C after incubation at 27 °C, resulting, also, in a slight increase in the HS-YFP quenching rate. As the fully-glycosylated form of WT-CFTR is a structurally stable protein with a low level of ubiquitination and PM retrieval [8,35,38,39], these differences may be related to compound efficacy in enhancing protein trafficking and stability. Indeed, our data point to a greater rescuing effect by VX-661 than by MCG1516A for WT-CFTR in the different experimental conditions (refer to Figure 4 and Figure 6).

To further examine the MoA of MCG1516A, we investigated its additivity to genetic revertants (G550E, R1070W, and 4RK), which partially rescue F508del-CFTR processing and PM expression by distinct mechanisms (Figure 7). In this context, G550E and R1070W are postulated to correct two distinct contact points in the three-dimensional structure of CFTR that are disrupted by F508del. G550E enables the formation of a salt bridge across the ATP binding site on NBD1 with amino acid residues from NBD2 [12], while R1070W reestablishes NBD1:ICL4 interaction by filling a pocket left empty by the absence of F508 residue at the NBD1 surface [13,15]. Our data suggest a putative binding site for MCG1516A on NBD1:NBD2 interface, as this compound was additive to R1070W in rescuing CFTR processing and PM expression, but not to G550E. Indeed, the lack of additivity of MCG1516A to low temperature and the genetic revertant G550E strongly suggests that this compound acts early in protein biosynthesis, i.e., before the nascent F508del-CFTR polypeptide chain is tagged for degradation. Similarly, VRT-325 was reported to be likely accommodated at the NBD1:NBD2 interface [13] and to stabilize F508del-CFTR at the early steps of co-translational folding [34]. This suggests that VRT-325 may be mechanistically close to MCG1516A, although VRT-325 was also additive to low temperature. In contrast to MCG1516A, VX-661 effects were additive to G550E, but not to R1070W, as previously also observed for VX-809 and RDR01752 [13,14]. Altogether, these data are consistent with MCG1516A and VX-661 having different binding sites on F508del-CFTR structure, a possibility further supported by their observed additive effects when co-administered (refer to Figure 1).

We also assessed additivity of MCG1516A with the 4RK revertant, which consists in the substitution of one arginine to lysine at each of the four retention/retrieval AFT motifs, thus allowing some F508del-CFTR protein to exit the ER [11]. Our biochemical and immunofluorescence results demonstrated that an increase by VX-661 in the amount of processed F508del-4RK-CFTR and expressed at the PM (Figure 7), as previously demonstrated for the similar drug VX-809 [13]. These observations suggest that this compound does not affect AFT-associated dominant ER retention, as also shown for RDR01752, VRT-325 and Corr4a [13,14], but in contrast to MCG1516A that reduced both F508del-4RK-CFTR processing and PM expression. A likely explanation for this behavior is the role of R555 (one of the four AFT mutated in 4RK) in the formation of NBD1:NBD2 dimer [40], the putative binding site of MCG1516A.

Besides the AFT retention motifs, CFTR exit from the ER depends on the presence of a diacidic code, which is disrupted in the DD/AA-CFTR variant without causing a folding defect [30]. Neither MCG1516A nor VX-661 rescued DD/AA processing and PM expressing, as also observed for VX-809, RDR01752, VRT-325, and Corr4a [13,14]. These findings demonstrate that MCG1516A is unable to overcome the lack of the Sec24-COPII-ER export signal, and its effects on F508del-CFTR could be increased if combined with a compound restoring DD/AA-CFTR traffic.

Because various reports have demonstrated that CF-causing mutations may respond differently to the same CFTR corrector, we also investigated here whether MCG1516A could rescue other CFTR trafficking mutants (i.e., class II), namely, G85E, L206W, I507del, V520F, R560S, M1101K, and N1303K (Figure 8). Of these, G85E, R560S, and N1303K are temperature-insensitive in contrast to F508del-CFTR [24,28,41]. In FRT cells, V520F, M1101K, and N1303K appeared to respond functionally to either MCG1516A or VX-661 in the FMP assay with greater effects when these compounds were combined. G85E only demonstrated a fairly small response when both compounds were used together. To further confirm these results, we performed WB analysis in CFBE cells expressing some of those CFTR mutations, as this is a more physiologically relevant cell model than FRT cells in the context of CF. Indeed, ER quality control machineries are different in CFBE and FRT cells, and in the latter, some mutant protein may evade ER-associated degradation, thus being more susceptible to corrector effects [31,32]. In CFBE cells, both MCG1516A and VX-661 led to the appearance of the fully-glycosylated form of L206W-CFTR with enhanced rescue promoted by the co-treatment. However, neither MCG1516A nor VX-661 rescued CFTR processing in G85E-, I507del-, R560S-, and N1303K-expressing cells. The absence of response of these mutants to several correctors have been previously reported [24,26,27,28], although recent studies demonstrated that the triple combination VX-445/VX-661/VX-770 rescues G85E and N1303K, albeit at significantly inferior levels of those observed for F508del-CFTR [25,42]. Because MCG1516A was also effective at increasing WT-CFTR processing and function, we tested its effects on certain CFTR mutants with residual function, in which there is reduced channel conductance without affecting protein trafficking (i.e., class IV), namely R334W, T338I, R347P, and S549N [43]. In FRT cells, both R334W and R347P responded to MCG1516A and VX-661 with greater responses promoted by the co-treatment, while T338I and S549R only responded to VX-661. WB analysis in CFBE cells confirmed the ability of MCG1516A and VX-661 to enhance R334W-CFTR processing, suggesting that this combination might be a feasible therapeutic option for individuals with CF having this mutation. Notably, R334W is not included in any list of CFTR mutations responsive to clinically approved modulator drugs [16]. Altogether, these data suggest that both L206W- and R334W-CFTR may be rescued by MCG1516A with greater efficacy when combined with VX-661. Despite such promising effects on heterologous systems, the effects of MCG1516A on L206W and R334W remain to be investigated in patient-derived material in order to provide a better prediction of the in vivo efficacy of this novel compound.

## 5. Conclusions

In summary, we found that MCG1516A rescues F508del-CFTR folding and trafficking to the PM, albeit at a lower efficacy than VX-661. MCG1516A was also effective in increasing WT-CFTR processing and PM expression. Despite its suboptimal effects, MCG1516A was able to rescue L206W- and R334W-CFTR processing and function in cell lines and had additive effects with VX-661, suggesting that this combination might be a feasible therapeutic option for individuals with CF having these rare mutants in addition to F508del. Our studies with low temperature and genetic revertants of F508del-CFTR indicate that MCG1516A and VX-661 act by distinct mechanisms (refer to Graphical abstract). Indeed, the data with revertants are consistent with a putative binding site of MCG1516A at the NBD1:NBD2 interface, while VX-661 appears to restore NBD1:ICL4 interaction. The observation that MCG1516A and VX-661 are not able to rescue DD/AA traffic suggests that there is still scope to further increase the correction of F508del-CFTR defects.

## Data Availability

The data presented here are available on request from the corresponding authors. The data are not publicly available due to privacy and ethical issues.

## Abbreviations

ABC, ATP-binding cassette; AFT, arginine-framed tripeptides; CF, cystic fibrosis; CFBE, cystic fibrosis bronchial epithelial; CFTR, cystic fibrosis transmembrane conductance regulator; ER, endoplasmic reticulum; ERQC, endoplasmic reticulum quality control; FBS, fetal bovine serum; FMP, FLIPR membrane potential; FRT, Fischer rat thyroid; Fsk, forskolin; Gen, genistein; HS-YFP halide sensitive yellow fluorescence protein; ICL, intracellular loop; MoA, mechanism of action; NBD, nucleotide-binding domain; PKA, protein kinase A; PKC, protein kinase C; RD, regulatory domain; TMD, transmembrane domain; WB, Western blot; WT, wild-type.
